# Molecular Diagnostics of Mucormycosis in Hematological Patients: A Literature Review

**DOI:** 10.3390/jof5040112

**Published:** 2019-11-29

**Authors:** Olga V. Shadrivova, Ekaterina V. Burygina, Nikolai N. Klimko

**Affiliations:** Department of Clinical Mycology, Allergy and Immunology, I. Mechnikov North Western State Medical University, Saint Petersburg 194291, Russia; olshadr@mail.ru (O.V.S.); katerina.prokura@gmail.com (E.V.B.)

**Keywords:** hematological patients, Mucorales, mucormycosis, molecular diagnosis, PCR, real-time quantitative PCR, sequencing

## Abstract

Objectives: to analyze the results of molecular methods applying for the diagnosis of mucormycosis in hematologic patients based on a literature review. Data sources: A systematic search in databases PubMed, Google Scholar for August 2019. Review eligibility criteria: original articles published in English, studies of molecular methods for the diagnosis of mucormycosis in hematologic patients. Results. We analyzed the research data from 116 hematological patients with mucormycosis, including children (6%). Patients with localized forms of mucormycosis prevailed (72%), and lung involvement was diagnosed in 58% of these cases. For molecular verification of the causative agent of mucormycosis, blood serum was most often used, less commonly postoperative and autopsy material, biopsy specimens, formalin-fixed paraffin-embedded samples and bronchoalveolar lavage, pleural fluid and sputum. The sensitivity of molecular diagnostics of mucormycosis in a cohort of hematological patients was 88.2%. Conclusion. The use of molecular techniques along with standard mycological methods will improve the diagnostics of mucormycosis in hematologic patients. However, prospective studies of the effectiveness of molecular methods for the diagnosis of mucormycosis of various etiologies in hematological patients, including children, using bronchoalveolar lavage (BAL) and cerebrospinal fluid (CSF) are needed.

## 1. Introduction

In recent decades the incidence of mucormycosis has been increasing due to the growth of the number of severely immunocompromised patients. In developed countries, mucormycosis is mostly seen in hematological patients [1,2]. Mucormycetes are the second most common causative agents of mycoses in hematological patients after the *Aspergillus* spp. and the third most common infection after candidiasis and aspergillosis in recipients of allogeneic hematopoietic stem cell transplants (allo-HSCT) [3,4]. At the same time, increased frequency of mucormycosis in hematological patients was noted. For example, in France from 1997 to 2006 in this cohort of patients was recorded an increasing incidence of this fungal infection for more than 24% per year, among allo-HSCT recipients—for more than 15% per year [5]. Mucormycosis in hematological patients differs from other groups of patients (with diabetes mellitus, trauma, etc.) in etiology, localization of infection and high mortality rate, which exceeds 50%. An increasing incidence of mucormycosis in hematological patients can be associated with the intensification of cytostatic chemotherapy and with the use of inactive against mucormycetes voriconazole for prevention and treatment of aspergillosis [6,7,8]. According to our previous studies [9], mucormycosis was diagnosed in 52% of patients within 1–225 days after the development of invasive aspergillosis. Meanwhile voriconazole was used in 74% of these patients for the treatment of aspergillosis.

According to this the role of early diagnosis of mucormycosis is growing, but the disease is often detected late, often postmortem [6,9,10]. The clinical and radiological signs are similar to other mold mycoses, for example aspergillosis [11]. Standard diagnostic tests, such as microscopy and culture, have low sensitivity. There are no standardized serological methods for the diagnosis of mucormycosis [6,7,8,11]. Histological examination is the “gold standard” of diagnosis but it is difficult to perform in this cohort of patients due to thrombocytopenia and severity of their condition. The use of new highly sensitive methods may be useful for the early diagnosis of mucormycosis in hematological patients [2,11].

Molecular methods are promising directions of modern laboratory diagnosis of fungal infections. The main advantages of these tests are high sensitivity and specificity, as well as the ability to use any substrates containing DNA for analysis [12].

Early diagnosis and timely initiation of mucormycosis treatment can improve the survival of patients and give a permit to continue the treatment of underlying hematologic disease. This will also reduce the time of hospitalization and, consequently, lower the treatment cost [11]. However, there are not enough publications on the effectiveness of molecular methods in the diagnosis of mucormycosis in hematological patients.

The aim of this study was to analyze the results of the application of molecular methods for the diagnosis of mucormycosis in hematological patients.

## 2. Materials and Methods

In a systematic search in the PubMed, Google Scholar databases we found 38 articles on molecular methods for the detection and identification of mucormycetes in various substrates. For the review we selected sources that met the following requirements: original articles in English, where oncohematological patients with diagnosed mucormycosis were included in the studies in accordance with the criteria of the European Organization for Research and Treatment of Cancer/Mycoses Study Group (EORTC/MSG, 15, June, 2008) [13].

The analysis did not include review articles, abstracts for conferences, descriptions of clinical cases, as well as articles where there were no characteristics of the background states of patients with mucormycosis, or the research results combined for various cohorts of patients, including non-hematological patients. The final search was carried out in August 2019.

## 3. Results and Discussion

The review included seven studies carried out in 2001–2017 [14,15,16,17,18,19,20] devoted to assessing the effectiveness of molecular methods for the diagnosis of mucormycosis in hematological patients. We analyzed the data from a survey of 116 oncohematological patients with mucormycosis presented in these articles (Table 1). The number of hematological patients in these studies was small and varied from five to 34 people. Among the examined patients, adults predominated (*n* = 110; 95%), there were significantly fewer children (*n* = 6; 5%). Meanwhile, in our register, children and adolescents accounted for 36% of all patients with mucormycosis [9]. Analysis of information on the treatment of background hematologic disease showed that approximately the same number of patients after cytostatic therapy and allo-HSCT were included in the studies, which coincides with the data from our register [9]. However, most studies were retrospective. Only in the Springer et al. 2016 study serum samples were collected and evaluated prospectively from 2008 to 2012 [16]. Therefore, additional prospective studies of the effectiveness of molecular methods for the diagnosis of mucormycosis in hematological patients, including children, are probably needed.

In all the works selected for this review, molecular diagnosis of mucormycosis was carried out using the polymerase chain reaction (PCR) method. Four studies used real-time PCR (RT PCR) for 18S rDNA [14,15,16,17]; in two studies, the 18S rDNA region was amplified using endpoint detection [18,19]. One study was based entirely on the determination of the sequence of internal transcribed spacer region (ITS) [20]. In a number of studies, DNA sequencing of either ITS or 18S rDNA regions was used to identify mucormycetes (Table 1).

In most cases (84%), molecular methods were used in patients with “proven” mucormycosis, according to the criteria of EORTC/MSG, 2008. The samples of patients with “possible” mycosis were less commonly used. In the study conducted by Springer et al. 2016 were included two oncohematological patients with unidentified mycosis, in whom mucormycetes were subsequently detected by RT-PCR. In addition, ten patients had a combination of mucormycosis with “probable” or “proven” aspergillosis, five patients were diagnosed with “probable” aspergillosis prior to molecular diagnosis of mucormycosis, and one patient was “proven” based on histological examination of *Fusarium* infection (Table 2). A combination of different types of mycoses is often detected in hematological patients. For example, according to our data, for 52% of patients mucormycosis was diagnosed 1–225 days after IA development [9].

Patients with localized forms of mucormycosis prevailed (*n* = 84, 72%), and lung involvement was diagnosed in 58% of these cases (*n* = 49; Table 2). Lesions of two or more organs were diagnosed in 26% (*n* = 30), rhinocerebral mucormycosis was in 9% of patients (*n* = 11) and isolated central nervous system damage was observed in one patient. This indicates the representativeness of the examined patients: lungs are the main localization of mucormycosis in hematological patients, rhinocerebral form and disseminated process are common [6,21]. According to a recent review, in allo-HSCT recipients CNS mucormycosis was diagnosed in 11% of all cases, in patients with malignancies—in 4–19% [22].

Analysis of the etiology of mucormycosis based on the results of substrate culture were possible only in 35 out of 82 cases (43%; Table 3). According to this, the number of hematological patients with mucormycosis of various etiologies was small: *Rhizopus* spp. = 11, *Lichtheimia* spp. = 10, *Rhizomucor* spp. = 6, *Mucor* spp. = 4 and *Cunninghamella* spp. = 3. The combination of two mucormycetes was detected in two patients.

Etiology of mucormycosis seemed to vary in different countries. For example, *Rhizopus* spp. (34%), *Mucor* spp. (19%) and *Lichtheimia* spp. (19%) were most commonly identified in patients with mucormycosis in Europe [8]. In comparison with these results the main pathogens of mucormycosis in Russia were *Rhizopus* spp. (47%), *Rhizomucor* spp. (28%) and *Lichtheimia corymbifera* (17%). According to our data, cultures from bronchoalveolar lavage (BAL) fluid and other samples were positive in 65% hematological patients with mucormycosis [9]. Probably, additional researches of effectiveness of molecular diagnostics for various etiology of mucormycosis including *Rhizomucor* spp., *Mucor* spp., *Cunninghamella* spp. and rare pathogens are needed.

Various materials were used for molecular verification of the causative agent of mucormycosis: most often blood serum, less often postoperative and autopsy material, biopsy specimens, histological paraffin blocks, BAL fluid, pleural fluid and sputum (Table 4). In the study performed by Scherer et al. 2018, several substrates (BAL, serum and histological samples) were examined at once.

The obtained data indicate that molecular methods for a blood serum study can be an important tool for the diagnosis of mucormycosis in hematological patients. Millon et al. in their study showed that mucormycetes DNA can be detected in at least 36/44 (81%) patients with “probable” or “proven” mucormycosis. Due to the retrospective design of the study, serum sampling and volume were not optimal, and the sensitivity level was 81%. However, with the exception of cases with insufficient serum volume for testing, the sensitivity of the molecular method reached 92% [15].

Springer et al. 2016 showed a high percentage of positive results when have being used RT-PCR for 18S rDNA to analyze sera from patients with “proven” and “probable” mucormycosis. Further sequencing revealed the DNA of two different pathogens of mucormycosis (*Lichtheimia* and *Rhizopu*s) and confirmed the pathogen identified by cultural methods in four out of five cases. *Rhizopus* spp. was also identified by this method in the case when the genus was not defined in the culture [16].

Bronchial lavage samples were examined in 25 patients, sputum and pleural fluid in three (Table 4). Scherer et al. 2018 showed that the sensitivity of molecular diagnostic tests for serum and BAL samples were comparable [17]. It was difficult to assess the effectiveness of molecular methods in sputum and pleural fluid for the diagnosis of mucormycosis due to the small number of patients included [20].

Since the main localization of mucormycosis in hematological patients are lungs, additional studies of the effectiveness of molecular diagnostic methods using BAL are probably needed.

We have not found the results of the cerebrospinal fluid (CSF) study, although rhinocerebral mucormycosis was in 9.5% of the examined patients. Probably, additional studies of the effectiveness of molecular diagnostic methods using CSF are also needed.

The results of Hammond et al. 2011’s studies showed that the detection of mucormycetes DNA by PCR in histological preparations (tissue fixed in paraffin was used in this work) could also be a useful tool for the diagnosis of mucormycosis. Of 27 tissue samples with initial histological signs of mucormycosis, PCR was positive in 22 samples. In addition, the researchers found a high level of correlation between the results of PCR diagnostics and culture: of 12 samples with a positive culture, mucormycosis was confirmed by PCR in 10 cases (83%), and results of sequencing corresponded to the result of culture identification to genus in nine samples. In addition, PCR diagnostics revealed molds in substrates of 12 patients in whom a fungal culture was not obtained [18].

Analysis of the summarized data as a result of our review indicates a high sensitivity of the molecular diagnostics of mucormycosis in a cohort of hematological patients. So, a positive PCR result was obtained in 98 cases of the 111 examined samples, thus the sensitivity was 88.2% (Table 5), while the culture sensitivity was 42.7% (Table 3).

As our literature analysis has shown, the spectrum of the methods of molecular diagnostics of mucormycosis used to date was mainly limited to RT-PCR and sequencing of 18S rDNA, as well as sequencing of the ITS region. DNA sequencing allowed us to get a slightly better taxonomic resolution, and RT-PCR met the criteria for a quick diagnostic method. In general, molecular diagnostic methods used in the studies we reviewed and intended for amplification of 18S rDNA allowed us to reach a sensitivity of 85–100%. The use of PCR identification of mucormycetes DNA in serum or BAL, along with standard mycological methods, can increase the effectiveness of early diagnosis of mucormycosis in hematological patients. At the moment an important problem is the lack of standardization of molecular methods. Fungal PCR Initiative (FPCRI)/Mucorales Lab working group of the International Society for Human and animal Mycology (ISHAM) are currently solving the questions about improving standardization and providing recommendations for Mucorales PCR assay, as was previously done for *Aspergillus* PCR assays by the European *Aspergillus* PCR Initiative (EAPCRI) working group [23,24].

## 4. Conclusions

1. Applying molecular methods along with standard mycological methods will improve the diagnosis of mucormycosis in hematologic patients.

2. Prospective studies of the effectiveness of molecular methods for the diagnosis of mucormycosis in hematological patients, including children, are needed.

3. Further researches on the effectiveness of the molecular diagnosis of mucormycosis of various etiologies, including *Rhizomucor* spp., *Mucor* spp., *Cunninghamella* spp. and rare pathogens are needed.

4. Since the main localizations of mucormycosis in hematological patients are lungs and the central nervous system, additional studies of the effectiveness of molecular diagnostic methods using BAL and CSF are needed.

## Figures and Tables

**Table 1:** Study design.

Author	Study Period	Number of Hemato-Logical Patients	Adults/Children	Chemotherapy/Allo-HSCT	Study Prospective/Retrospective	Molecular Diagnostic Method	Target
Millon 2013 [14]	2004–2012	7	6/1	6/1	retrospective	real-time PCR	18S rDNA
Millon 2016 [15]	2012–2014	34	32/2	21/13	retrospective	real-time PCR	18S rDNA
						sequencing	18S rDNA ITS
Springer 2016 [16]	2010–2014	12	12/0	ND/ND	prospective	real-time PCR	18S rDNA
						sequencing	
Scherer 2018 [17]	2013–2017	20	20/0	12/8	retrospective	real-time PCR	18S rDNA
Hammond 2011 [18]	2001–2009	29	29/0	14/15	retrospective	PCR	18S rDNA
						sequencing	
Gholinejad-Ghadi 2018 [19]	2004–2007; 2015–2017	5	3/2	ND/ND	retrospective	PCR	18S rDNA
						sequencing	18S rDNA region ITS
Guinea 2017 [20]	2007–2015	9	8/1	6/3	retrospective	PCR	region ITS
						sequencing	
Total:		116	110/6	59/40			

ND: no data.

**Table 2:** Characteristics of mucormycosis in hematological patients.

Author	Number of Hemato-Logical Patients	Proven/Probable Mucormycosis	Possible/Unidentified Mycosis	Including: Mixed Infection (Mucor-Mycosis + Invasive Aspergillosis)	Other Mycoses	Isolated Mucor-Mycosis: Lung/Others	2 and > Organs/Rhino-Cerebral
Millon 2013	7	7/-	-	1	-	1/-	4/2
Millon 2016	34	18/16	-	6	ND	16/2	11/5
Springer 2016	12	5 ^a^	5/2	-	-	1/3 ^e^	1/-
Scherer 2018	20	5/3	7	2	4 ^b/^1 ^c^	19/1 ^d^	-
Hammond 2011	29	29/-	-	-	-	6/11	12/-
Gholinejad-Ghadi 2018	5	5/-	-	-	-	-	1/4
Guinea 2017	9	4/5	-	1 ^b^	-	6/2	1/-
Total:	116	97	14	10	5	49/19	30/11

^a^ cases of “proven” or “probable” mucormycosis. ^b^ “probable” invasive aspergillosis; ^c^ “proven” fusariosis; ^d^ central nervous system (CNS); ^e^ no localization data in seven patients. ND: no data.

**Table 3:** Etiology of mucormycosis in hematological patients.

Author	Number of Hemato-Logical Patients	Positive Culture (Number)	*Rhizopus* spp.	*Mucor* spp.	*Cunninghamella* spp.	*Rhizomucor* spp.	*Lichtheimia* spp.	2 or More Mucormycetes
Millon 2013	7	5	2	0	0	0	3	
Millon 2016	NA ^b^	NA	NA	NA	NA	NA	NA	NA
Springer 2016	12	5	2	1	0	0	2	0
Scherer 2018	20	3	0	0	0	2	1	
Hammond 2011	29	13	5	3	2	2	1	1
Gholinejad-Ghadi 2018	5	1 ^a^	1	0	0	0	0	
Guinea 2017	9	8 ^c^	1	0	1	2	3	-
Total:	82	35	11	4	3	6	10	1

^a^ no data for other patients; ^b^ analysis of the etiology of mucormycosis based only on the cultural method was not possible. ^c^ one of eight isolates was not available for study.

**Table 4:** Samples used for molecular diagnostics.

Author	Number of Hematological Patients	Samples	Histology, Biopsy Specimens	BAL	Sputum, Pleural Fluid	Serum
Millon 2013	7	tissue (fresh or paraffin-embedded)	7	-	-	-
Millon 2016	34 ^a^	serum	-	-	-	34 ^a^
Springer 2016	12 ^b^	serum	-	-	-	12
Scherer 2018	20	histology; BAL, serum	6	20	-	19
Hammond 2011	29	histology, tissue aspirates, autopsy specimens, biopsy specimens	29 ^c^	-	-	-
Gholinejad-Ghadi 2018	5	formalin-fixed paraffin-embedded samples	5	-	-	-
Guinea 2017	9	BAL/bronchial secretions, pleural fluid, sputum, biopsy tissue	4	5	3	-
Total:	116		51	25	3	65

^a^ 1 and > serum samples were obtained from each of 34 patients. ^b^ the study included only hematological patients in whom species identification was performed using molecular diagnostic methods. ^c^ including five autopsy samples.

**Table 5:** Cultures and PCR results.

Author	Number of Hematological Patients	PCR Was Performed	PCR (+) Total	PCR (+)/Cultures (+)	PCR (–)/Cultures (+)	PCR (+)/Cultures (–) or Unavailable	PCR (–)/Cultures (–) or Unavailable
Millon 2013	7	7	7	5/5	0	2/2	-
Millon 2016	34	34	30	NA	NA	NA	-
Springer 2016	12	12	12	5/5	0	7/7	-
Scherer 2018	20	20	20 ^a^	3/3	0	17/17	-
Hammond 2011	29	27	22	10/12	2/12	12/15	3/15
Gholinejad-Ghadi 2018	5	5	4	1/1	0	3/4	1/1
Guinea 2017	9	6	3	2/5	3/5	1/1	-
Total:	116	111	98	26/31	5/17	42/46	

^a^ PCR assay was positive in bronchoalveolar lavage (BAL) and/or serum in all patients. NA: not applicable.
